# Reduction of Quinones by NADH Catalyzed by Organoiridium Complexes**

**DOI:** 10.1002/anie.201300747

**Published:** 2013-03-07

**Authors:** Zhe Liu, Robert J Deeth, Jennifer S Butler, Abraha Habtemariam, Mark E Newton, Peter J Sadler

**Affiliations:** Department of Chemistry, University of WarwickCoventry, CV4 7AL (UK) E-mail: p.j.sadler@warwick.ac.uk; Department of Physics, University of WarwickCoventry, CV4 7AL (UK)

**Keywords:** hydride transfer, iridium, NADH, quinones, radical reactions

Quinones (Q) are a class of fully conjugated cyclic dione compounds that are widely distributed in nature.^1^ They can function as electron carriers in electron-transport chains (e.g. aerobic respiration)^2^ and undergo one- or two-electron reduction, coupled with protonation, leading to the corresponding semiquinones (QH^.^) or hydroquinones (QH_2_), respectively (Scheme 1). In biological systems, quinones are often reduced by coenzyme NAD(P)H (Scheme 2) in the presence of cellular reductases, such as NADH ubiquinone oxidoreductase, NADH cytochrome b_5_, and NADPH cytochrome P-450 reductase.^3^ Apart from enzymatic methods, the reduction of quinones by chemical, photochemical, and electrochemical methods has been investigated because of its biological and industrial importance.^4^ However, there are only a few reports of metal involvement in the reduction of quinones.^5^ For example, Sc^3+^ can form metal–quinone radical adducts using the cyclometalated iridium complex [Ir(ppy)_3_] (ppy=2-phenylpyridine) as an electron donor.5a Organometallic iridium complexes have potential for application in catalysis^6^ and biology.^7^ Coenzyme NADH can transfer hydride ions to cyclopentadienyl–Ir^III^ complexes, generating iridium–hydride complexes.^8^ Here we investigate whether cyclopentadienyl–Ir^III^ complexes can use NADH as a hydride source for the reduction of quinones, thus mimicking the action of reductases. We chose quinones vitamin K_3_ (menadione) and the related duroquinone as substrates (Scheme 2), because vitamin K is required for the synthesis of certain proteins that are involved in blood coagulation and metabolic pathways in bones and other tissues,^9^ and may play an important role in the treatment of cancer, Alzheimer’s and other diseases.^10^ This appears to be the first report of the catalytic reduction of quinones by an organometallic complex, and intriguingly, appears to involve the unusual Ir^II^ oxidation state in a novel mechanism.

**Scheme 1:**
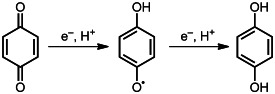
One- and two-electron reduction of a quinone (e.g. benzoquinone) leading to semiquinone and hydroquinone, respectively.

**Scheme 2:**
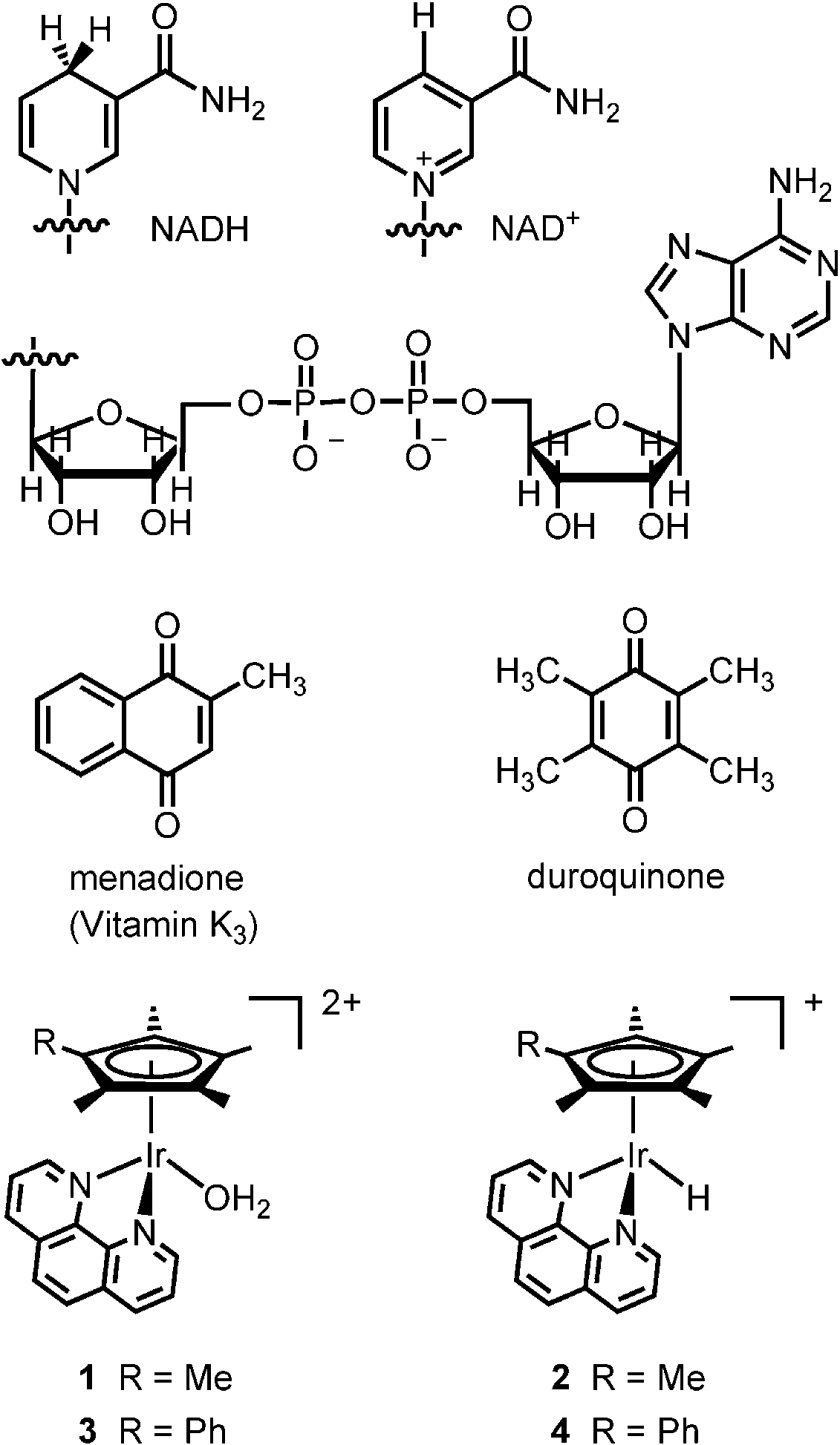
Structures of NADH, NAD^+^, quinones, and Ir^III^ complexes studied herein.

Complexes [(η^5^-C_5_Me_5_)Ir(phen)(H_2_O)]^2+^ (**1**, phen=1,10-phenanthroline) and [(η^5^-C_5_Me_4_C_6_H_5_)Ir(phen)(H_2_O)]^2+^ (**3**; Scheme 2) were synthesized as PF_6_ salts and characterized by ^1^H NMR spectroscopy, ESI-MS, and CHN elemental analysis (see the Supporting Information for details).

First we studied the interactions of NADH, [(η^5^-C_5_Me_5_)Ir(phen)(H_2_O)]^2+^ (**1**) and menadione by ^1^H NMR spectroscopy. Upon the addition of two molar equivalents of NADH to **1** (1 mm) in [D_4_]MeOD/H_2_O (1:9), the colour of the solution changed from light to dark yellow immediately. The ^1^H NMR spectrum recorded at 298 K showed a sharp singlet at −11.3 ppm within the first 10 min, which is assignable to the iridium–hydride complex **2** (Scheme 2) together with a new set of signals that are attributable to NAD^+^ (Figure 1 b). When one molar equivalent of menadione was added to the reaction mixture, the resonances corresponding to **2** disappeared and were replaced by signals for the aqua complex **1** (Figure 1 c). No sharp signals for the added menadione were observed, only a set of broad resonances (Figure 1 c), thus suggesting that the menadione might be present as a paramagnetic species.

**Figure 1:**
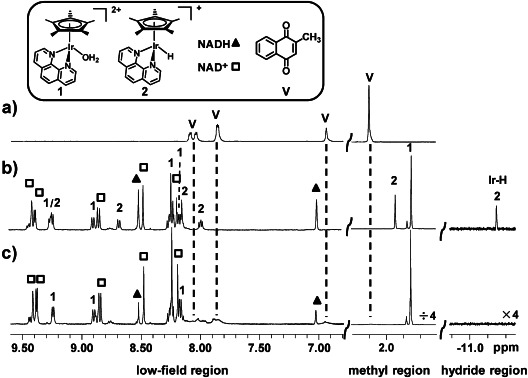
^1^H NMR spectra showing the formation of complex **2** [(η^5^-C_5_Me_5_)Ir(phen)(H)]^+^ and its reaction with menadione in [D_4_]MeOD/H_2_O (1:9) at 298 K. a) Menadione alone. b) Formation of hydrido complex **2** from reaction of [(η^5^-C_5_Me_5_)Ir(phen)(H_2_O)]^2+^ (**1**; 1 mm) with NADH (2 mm). c) Disappearance of signals of hydride **2** and broadening of signals of menadione 10 min after addition of menadione (1 mm) to mixture in (b).

To investigate the formation of paramagnetic products, EPR studies were carried out. Addition of menadione (1 mm) to a solution containing NADH (0.5 mm) and complex **1** (160 μm) in phosphate buffer (pH 7.2) resulted in an EPR spectrum that is consistent with the formation of the menadione semiquinone radical anion (M^.−^; Figure 2 a), whereas no EPR signal was detected in the absence of any of the three reactants. The simulated spectrum is characteristic of the menadione semiquinone radical anion (M^.−^) with hyperfine coupling constants a^H^_H5=H6=H7=H8_=3.03 G, a^H^_H3_=0.65 G (Figure 2 b), which agree with a previous report.^11^ The menadione radical anion formed here was stable for more than 20 h. Although some metal–quinone radical adducts have been reported,5a so far no superhyperfine splitting as a result of coupling with iridium nuclei was detected. Integration of the EPR signals (relative to a standard tempol (4-hydroxy-2,2,6,6-tetramethylpiperidine-1-oxyl) solution) showed that almost all the menadione (93 %) had been converted to menadione semiquinone.

**Figure 2:**
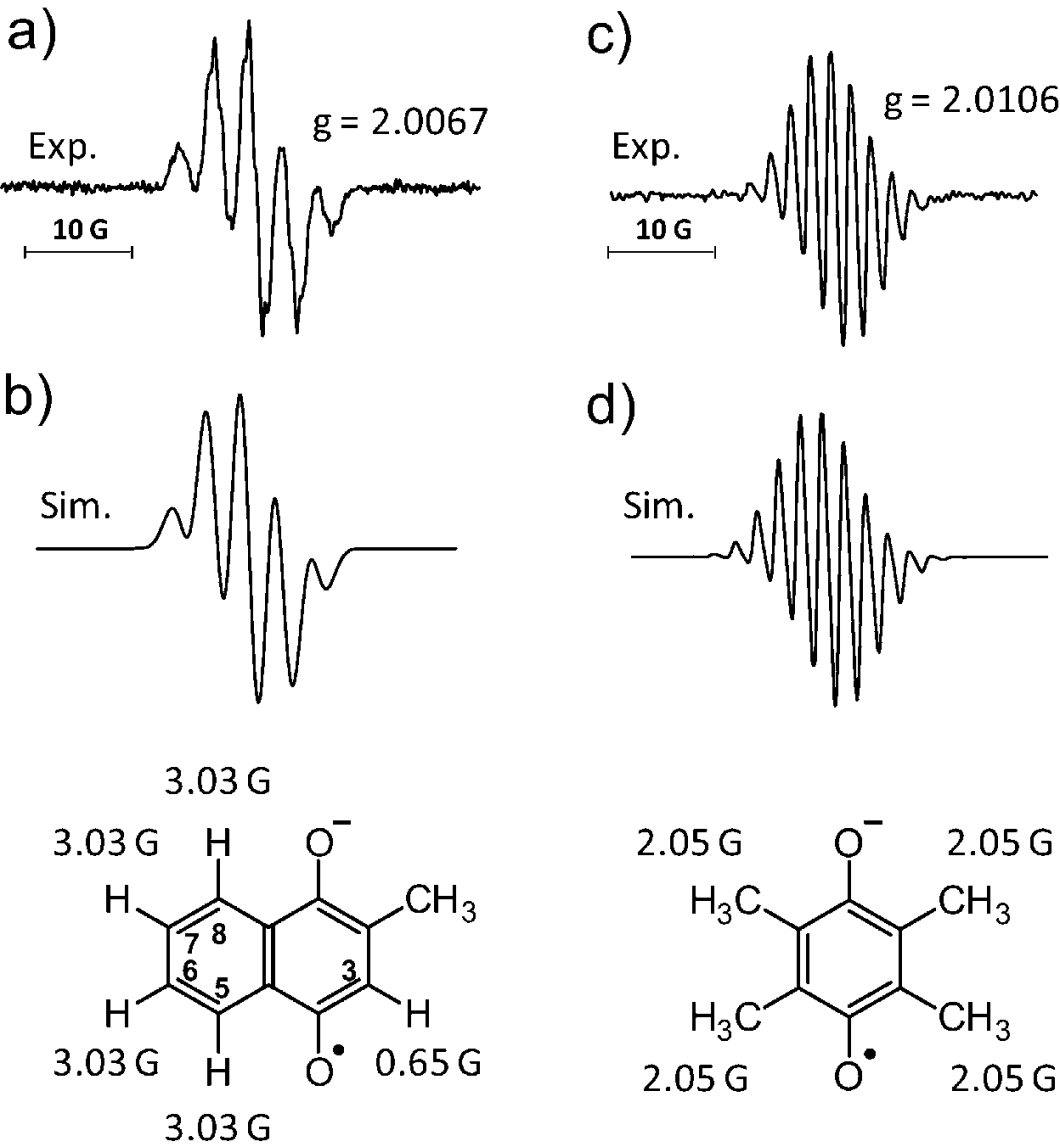
X-band EPR spectra at around 290 K. a) Menadione radical anion (M^.−^) generated by the reduction of menadione (1 mm) by NADH (0.5 mm) catalyzed by complex **1** or **3** (160 μm) in phosphate buffer (pH 7.2, 9 h). b) Simulated M^.−^ EPR spectrum with hyperfine coupling constants. c) Durosemiquinone radical anion (D^.−^) generated by reduction of duroquinone (2 mm) by NADH (1 mm) catalyzed by complex **1** or **3** (330 μm) in phosphate buffer (pH 7.2, 10 h). d) Simulated D^.−^ EPR spectrum with hyperfine coupling constants.

Strikingly, these data show the turnover of more than one molar equivalent of M^.−^ per Ir^III^, thus suggesting that complex **1** can act as a catalyst. The catalytic reduction of menadione was further studied by EPR spectroscopy. Various molar excesses of menadione and NADH were added to a solution of complex **1** in phosphate buffer (pH 7.2; entries 1–4 in Figure 3) and EPR spectra were recorded. The turnover numbers (TON) and initial turnover frequencies (TOF, expressed as the number of moles of radical formed per mole of Ir^III^ after the reaction was run for one hour) of these reactions increased with the concentrations of NADH and menadione (Figure 3). The maximum TON of 56.6 was observed for the reaction in entry 3 (molar ratio=1:30:80), and the maximum TOF of 12.4 h^−1^ for the reaction in entry 4 (1:60:120). Notably, a comparison of the TON with the amount of NADH present in entries 1–3 suggests that every molecule of NADH can reduce two quinone molecules (i.e. the semiquinone radical is formed). In entry 4, a similar TON was attained as in entry 3, although the concentrations of NADH and VK_3_ were increased, indicating that hydride transfer from NADH to **1** had reached saturation.

**Figure 3:**
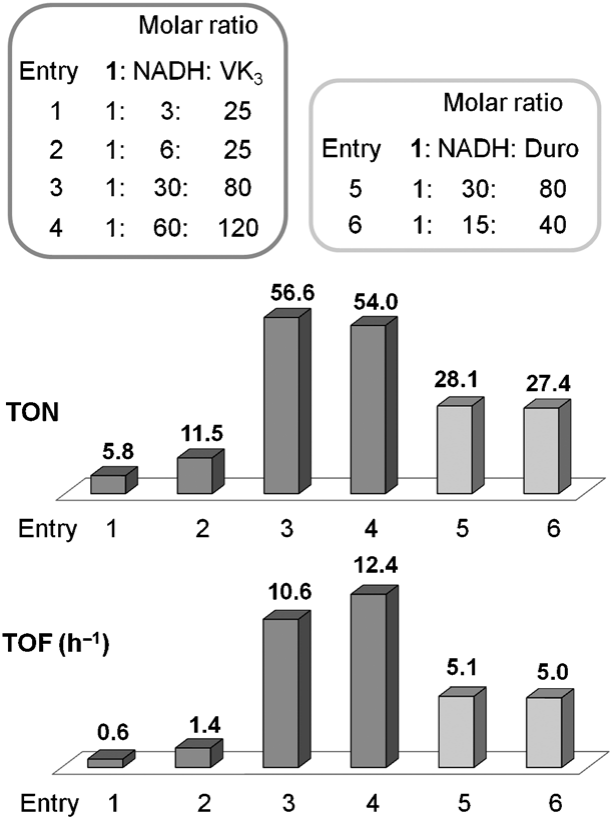
Reduction of menadione (VK_3_, entries 1–4) and duroquinone (Duro, entries 5 and 6) by NADH catalyzed by complex **1** (80 μm).

Similar NMR (Figure S1 in the Supporting Information) and EPR results (Figure 2 c and d) were obtained for the catalytic reduction of duroquinone by NADH/complex **1** (entries 5 and 6 in Figure 3), with a maximum TON of 28.1 and a maximum TOF of 5.1. These values are about half those for the reduction of menadione (entry 3). The presence of three additional electron-donating methyl groups makes duroquinone more difficult to reduce, which is in line with reported half-wave potentials.^12^ As with menadione, one mole of NADH reduces two moles of duroquinone (entry 6).

We also studied the reduction of quinones by NADH/[(η^5^-C_5_Me_4_C_6_H_5_)Ir(phen)(H_2_O)]^2+^ (**3**). The reaction pathways were similar to those of complex **1** (Figure 2, and Figure S2 in the Supporting Information), showing that the phenyl substituent on the Cp* has little effect on the reaction.

Since NADH is effectively a two-electron (hydride) donor, it is surprising that the reduction of quinone appears to involve two quantitative one-electron donor steps to give the semiquinone product. Although one-step hydride transfer from NADH and analogues to quinones (NADH+Q→NAD^+^+QH^−^), and multi-step hydride transfer (electron transfer followed by proton/electron transfer, e^−^+H^+^+e^−^) have been previously described,^13^ no mechanism for the reduction of quinone by metal-based hydrides has been reported.

DFT calculations (see the Supporting Information for details) were used to characterize various possible stationary points to provide insight into the likely mechanism of the iridium-catalyzed reduction of these quinones. A possible mechanism for the catalytic reduction of quinone to semiquinone by NADH/complex **1** is shown in Scheme 3 a. In the first step, a hydride ion is transferred from NADH to complex **1** to form the iridium–hydride complex **2** and NAD^+^. One-electron transfer to the quinone together with proton transfer to the phosphate buffer then generates the deprotonated semiquinone radical (Q^.−^) and a transient Ir^II^ center. Next, an electron is transferred from Ir^II^ to a second quinone molecule, resulting in further formation of semiquinone radical anion (Q^.−^) and regeneration of Ir^III^. The assumed formation of semiquinone radical anions is based on their relatively low p*K*_a_ value (ca. 5).^14^ Subsequent coordination of a water molecule to the Ir^III^ center completes the catalytic cycle. This oxidation state (II) is unusual for iridium and there are only a few reports of Ir^II^.^15^

**Scheme 3:**
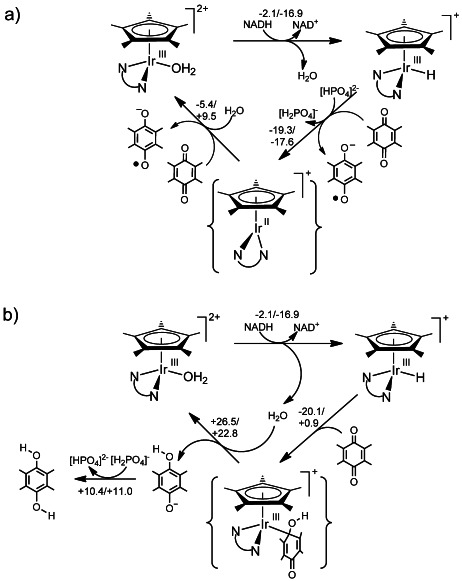
Two possible mechanisms for the catalytic reduction of duroquinone by complex **1**. a) Reduction through two sequential one-electron transfers. Figure S3 in the Supporting Information shows a plot of the singly occupied molecular orbital of the Ir^II^ d^7^ complex. b) Reduction through a single two-electron transfer. The pairs of numbers associated with each step are potential-energy/free-energy couples (in kcal mol^−1^). The semiquinone has a p*K*_a_ of around 5 and is therefore negatively charged at pH 7.2, whereas the quinol is protonated (p*K*_a_ higher than 11).^14^

The results of DFT calculations on an alternative two-electron reduction pathway, which would give rise to the fully reduced dihydroquinone, are shown in Scheme 3 b. The mechanism involves a one-step hydride transfer from complex **2** to the quinone via an intermediate with an Ir–C(quinone) bond. In this case, the quinol is released and protons are taken up from the buffer because of the higher p*K*_a_ value of the dihydroquinol.

In both cases, the regeneration of the aqua complex is the critical step. In Scheme 3 a, this step appears favored on the potential-energy surface, but disfavored on the free-energy surface, and in Scheme 3 b, this steps appears to be strongly disfavored on both surfaces. Thus, notwithstanding the difficulties of modeling H^+^/e^−^ transfers and the need to invoke the phosphate buffer to balance the various reaction steps, the DFT calculations generally support two consecutive one-electron transfers (pathway in Scheme 3 a), generating the transient Ir^II^ complex and the experimentally-detected semiquinone. The alternative dihydroquinone pathway (Scheme 3 b), in which only Ir^III^ species are involved, appears not to be viable theoretically.

In conclusion, organometallic cyclopentadienyl–iridium complexes offer the prospect of carrying out catalytic reductions of quinones without an enzyme through hydride transfer from NADH. These reactions produce semiquinones rather than the two-electron reduced products, the dihydroquinones. DFT calculations suggest that the reactions occur through a novel mechanism that involves an unusual transient Ir^II^ state, in which the iridium center plays a key role in promoting this one-electron pathway. This appears to be the first report of a catalytic reduction of a quinone by an organometallic complex, and may have promising potential for the design of catalytic metallodrugs and for activation of electron transfer in biological systems.^16^ Such organometallic complexes may therefore be valuable for the modulation of the redox status of cells (a potential drug target), as enzyme mimics, and for biocoupled hydrogenation reactions.

## Supplementary material

As a service to our authors and readers, this journal provides supporting information supplied by the authors. Such materials are peer reviewed and may be re-organized for online delivery, but are not copy-edited or typeset. Technical support issues arising from supporting information (other than missing files) should be addressed to the authors.

## References

[1] Dey PM, Dey PM, Harborne JB (1989). Methods in Plant Biochemistry.

[2a] Soballe B, Poole RK (1999). Microbiology.

[2b] Hirst J (2005). Biochem. Soc. Trans.

[2c] Yagi T, Matsuno-Yagi A (2003). Biochemistry.

[3a] Matsushita K, Ohnishi T, Kaback HR (1987). Biochemistry.

[3b] Schenkman JB, Jansson I (2003). Pharmacol. Ther.

[3c] Bachur NR, Gordon SL, Gee MV, Kon H (1979). Proc. Natl. Acad. Sci. USA.

[3d] Li R, Bianchet MA, Talalay P, Amzel LM (1995). Proc. Natl. Acad. Sci. USA.

[3e] Hirst J (2010). Biochem. J.

[4a] Guin PS, Das S, Mandal PC (2011). Int. J. Electrochem.

[4b] Müh F, Glöckner C, Hellmich J, Zouni A (2012). Biochim. Biophys. Acta Bioenerg.

[4c] Haroon Y, Bacon DS, Sadowski JA (1987). Biomed. Chromatogr.

[4d] Abraham I, Joshi R, Pardasani P, Pardasani RT (2011). J. Braz. Chem. Soc.

[5a] Yuasa J, Suenobu T, Fukuzumi S (2006). ChemPhysChem.

[5b] Fukuzumi S, Okamoto T (1993). J. Am. Chem. Soc.

[6a] Oro LA, Claver C (2008). Iridium Complexes in Organic Synthesis.

[6b] Andersson PG (2011). Iridium Catalysis.

[7a] Liu Z, Habtemariam A, Pizarro AM, Fletcher SA, Kisova A, Vrana O, Salassa L, Bruijnincx PCA, Clarkson GJ, Brabec V, Sadler PJ (2011). J. Med. Chem.

[7b] Liu Z, Habtemariam A, Pizarro AM, Clarkson GJ, Sadler PJ (2011). Organometallics.

[7c] Liu Z, Salassa L, Habtemariam A, Pizarro AM, Clarkson GJ, Sadler PJ (2011). Inorg. Chem.

[7d] Kastl A, Wilbuer A, Merkel AL, Feng L, Di Fazio P, Ocker M, Meggers E (2012). Chem. Commun.

[7e] Lo KK-W, Li SP-Y, Zhang KY (2011). New J. Chem.

[7f] Schäfer S, Sheldrick WS (2007). J. Organomet. Chem.

[8] Betanzos-Lara S, Liu Z, Habtemariam A, Pizarro AM, Qamar B, Sadler PJ (2012). Angew. Chem.

[b01] (2012). Angew. Chem. Int. Ed.

[9a] Weber P (2001). Nutrition.

[9b] Suttie JW (1995). Annu. Rev. Nutr.

[10a] Saxena SP, Israels ED, Israels LG (2001). Apoptosis.

[10b] Nimptsch K, Rohrmann S, Linseisen J (2008). Am. J. Clin. Nutr.

[10c] Allison AC (2001). Med. Hypotheses.

[11] Kubata BK, Kabututu Z, Nozaki T, Munday CJ, Fukuzumi S, Ohkubo K, Lazarus M, Maruyama T, Martin SK, Duszenko M, Urade Y (2002). J. Exp. Med.

[12] Frontana C, Vázquez-Mayagoitia Á, Garza J, Vargas R, González I (2006). J. Phys. Chem. A.

[13a] Gȩbicki J, Marcinek A, Zielonka J (2004). Acc. Chem. Res.

[13b] Zhu X-Q, Yang Y, Zhang M, Cheng J-P (2003). J. Am. Chem. Soc.

[13c] Yuasa J, Yamada S, Fukuzumi S (2008). Angew. Chem.

[b02] (2008). Angew. Chem. Int. Ed.

[13d] Yuasa J, Yamada S, Fukuzumi S (2006). J. Am. Chem. Soc.

[14] Rich PR, Bendall DS (1980). Biochim. Biophys. Acta Bioenerg.

[15a] Hetterscheid DGH, de Bruin B (2006). J. Mol. Catal. A.

[15b] Cheung CW, Chan KS (2011). Organometallics.

[15c] Kimura T, Ishiwata K, Kuwata S, Ikariya T (2012). Organometallics.

[15d] Takahashi Y, Fujita K-i, Yamaguchi R (2008). Eur. J. Inorg. Chem.

[15e] Meiners J, Scheibel MG, Lemée-Cailleau M-H, Mason SA, Boeddinghaus MB, Fässler TF, Herdtweck E, Khusniyarov MM, Schneider S (2011). Angew. Chem.

[b03] (2011). Angew. Chem. Int. Ed.

[15f] Teets TS, Cook TR, McCarthy BD, Nocera DG (2011). Inorg. Chem.

[16a] Sasmal PK, Streu CN, Meggers E (2013). Chem. Commun.

[16b] Cowan JA (2008). Pure Appl. Chem.

[16c] Hocharoen L, Cowan JA (2009). Chem. Eur. J.

[16d] Kuznetsov AM, Ulstrup J (1999). Electron transfer in chemistry and biology: an introduction to the theory.

