# Supplementary material for: Reduction of Quinones by NADH Catalyzed by Organoiridium Complexes
Source: Angew Chem Int Ed Engl. 2013 Mar 7;52(15):4194–7. doi: 10.1002/anie.201300747 (PMC3660790; doi:10.1002/anie.201300747)
Supplement: Supplementary file 1 [file anie0052-4194-SD1.pdf]

Angewandte Chemie
Eine Zeitschrift der Gesellschaft Deutscher Chemiker

Supporting Information
© Wiley-VCH 2013
69451 Weinheim, Germany

Reduction of Quinones by NADH Catalyzed by Organoiridium Complexes**

*Zhe Liu, Robert J. Deeth, Jennifer S. Butler, Abraha Habtemariam, Mark E. Newton, and Peter J. Sadler**

anie_201300747_sm_miscellaneous_information.pdf

Supporting information

Materials and Synthesis S2

Methods and Instrumentation S3-S5

Figures S1-S3 S6-S8

Materials and Synthesis

β-Nicotinamide adenine dinucleotide reduced dipotassium salt (NADH), menadione, duroquinone, and 4-hydroxy-2,2,6,6-tetramethylpiperidine 1-oxyl (Tempol) were obtained from Sigma-Aldrich. The solvents used for ^{1}H NMR spectroscopy were methanol-d_4 from Aldrich, and deionized water. Quartz tubes (1.0 mm × 1.2 mm O.D) for EPR studies were purchased from Wilmad Labglass.

General Procedure for the Synthesis of the Complexes. Solutions containing 1 mol equiv of the appropriate dimer $[(\eta^5\text{-}C_5Me_5)IrCl_2]_2$ or $[(\eta^5\text{-}C_5Me_4C_6H_5)rCl_2]_2$, 4 mol equiv of $AgNO_3$ in MeOH (10 mL) and water (20 mL) were stirred at ambient temperature for 24 h. The precipitate (AgCl) was removed by filtration through a glass wool plug, and 2 mol equiv of 1,10-phenanthroline were added to the filtrate. The reaction mixture was stirred at ambient temperature for 12 h. The volume was slowly reduced to half on a rotary evaporator and 10 mol equiv of NH_4PF_6 was added. The yellow precipitate that formed was collected by filtration, washed with diethyl ether, and recrystallized from methanol/diethyl ether.

$[(\eta^5\text{-}C_5Me_5)Ir(phen)(H_2O)](PF_6)_2$ (1). Yield: 66%. ^{1}H NMR(DMSO-d_6): δ = 9.36 (d, 2H, J = 5.2 Hz), 8.89 (d, 2H, J = 8.0 Hz), 8.37 (s, 2H), 8.32 (dd, 2H, J = 5.3, 5.5 Hz), 1.72 (s, 15H). Anal. Calcd for $C_{22}H_{25}F_{12}IrN_2OP_2$ (815.59): C, 32.40; H, 3.09; N, 3.43. Found: C, 32.61; H, 3.02; N, 3.56. MS: m/z 254.1 $[M – H_2O]^{2+}$.

$[(\eta^5\text{-}C_5Me_4C_6H_5)Ir(phen)(H_2O)](PF_6)_2$ (3). Yield: 60%. ^{1}H NMR (acetone-d_6): δ = 9.49 (d, 2H, J = 5.2 Hz), 9.08 (d, 2H, J = 8.3 Hz), 8.44 (s, 2H), 8.30 (dd, 2H, J = 5.3, 5.3 Hz), 7.57 (m, 5H), 1.99 (s, 6H), 1.83 (s, 6H). Anal. Calcd for $C_{27}H_{27}F_{12}IrN_2OP_2$ (877.66): C, 36.95; H, 3.10; N, 3.19. Found: C, 37.23; H, 3.00; N, 3.26. MS: m/z 285.1 $[M – H_2O]^{2+}$.

Methods and Instrumentation

Mass Spectrometry. Electrospray ionization mass spectra (ESI-MS) were obtained on a Bruker Esquire 2000 Ion Trap Spectrometer. Samples were prepared in CH_3CN/H_2O (1:1 v/v). The mass spectra were recorded with a scan range of *m/z* 50–1000 for positive ions.

Elemental Analysis. CHN elemental analyses were carried out on a CE-440 elemental analyzer by Exeter Analytical (UK) Ltd.

NMR Spectroscopy. ^{1}H NMR spectra were acquired in 5 mm NMR tubes at 298 K (unless otherwise stated) on either Bruker DRX-500, Bruker AV III 600 or Bruker AV II 700 NMR spectrometers. All data processing was carried out using XWIN-NMR version 3.6 (Bruker U.K. Ltd.). ^{1}H NMR chemical shifts were internally referenced to methanol-d_4 (3.33 ppm) for aqueous solutions. For ^{1}H NMR experiments 90% H_2O/10% MeOD-d_4 was used as solvent, to allow locking and minimize H/D exchange of the Ir-H species. The water peak was suppressed using presaturation or *SHAKA* excitation sculpting with gradients.[1]

Electron Paramagnetic Resonance. EPR spectra were recorded at ambient temperature (*ca.* 290 K) on a Bruker EMX (X-Band) spectrometer. A cylindrical Tm_{110} mode cavity (Bruker 4103TM) was used. This cavity is particularly useful for studies of aqueous or other samples exhibiting high dielectric loss. The samples were transferred to spectrosil quartz tubes with inner diameter of 1.0 mm and outer diameter of 1.2 mm sealed with T-Blu Tac®. This tube was placed inside a larger quartz tube (outer diameter 2.0 mm) so that the sample could be accurately and reproducibly positioned inside the resonator. Typical key EPR spectrometer settings were modulation amplitude 2.0 G and microwave power 0.63 mW, 5.0 $\times 10^4$ receiver gain, sweep time 20.97 s (durosemiquinone radical) or 41.94 s (menadione radical) with repeated number of 10 X-scans, resolution in X is 1024 (durosemiquinone radical) or 2048 (menadione radical). Spectra were analysed using the Bruker WINEPR software. The

concentrations of radicals were determined from a standard calibration curve based on known concentrations of the EPR standard Tempol.

pH Measurements. pH values were measured at ambient temperature using a Corning 240 pH meter equipped with a micro combination KNO_3 (chloride-free) electrode calibrated with Aldrich buffer solutions of pH 4, 7, and 10.

DFT Calculations. All DFT calculations used the ORCA program suite, version 2.9.1.[2] Since we were only interested in establishing the general viability of the two proposed mechanistic pathways, only local minima structures were located. Moreover, given the experimental pK_a of semiquinone radicals and quinols, the former were assumed to be deprotonated (hence mono anions) while the latter was assumed fully protonated (hence neutral). Since the reactions were run in a phosphate buffer, suitable phosphate species were employed in order to balance charges and protonation states. All geometries were optimized using the Becke-Perdew functional (BP86[3]) without any symmetry constraints using the relativistic zeroth order regular approximation (ZORA), ZORA def2-SVP basis sets[4] and the resolution of identity approximation. Grimme's 2006 empirical dispersion correction[5] was applied and numerical frequencies were computed both to confirm that each structure corresponded to a local minimum as well as to enable the estimation of the free energy, the latter obtained via the standard statistical mechanical methods implemented in ORCA. Unrestricted Kohn-Sham DFT was used for the open shell systems. Although of dubious significance in DFT, the computed spin operator expectation value $<S^2>$ never deviated significantly from the formal S=1/2 value of 0.75.

^{1}H NMR of Reactions with Menadione. NADH (2 mol equiv) was added to an NMR tube containing a 1 mM solution of $[(\eta^5\text{-}C_5Me_5)Ir(phen)(H_2O)]^{2+}$ (**1**) in 10% MeOD-d_4/90% H_2O at ambient temperature. ^{1}H NMR spectra of the resulting solutions were recorded at 298 K. After 15 min of reaction, an equimolar amount of menadione (1 mM) was added to the above solution, and the ^{1}H NMR spectra of the resulting solutions were recorded at 298 K.

EPR of Reactions with Menadione. Menadione (1 mM) was added to the solution containing NADH (0.5 mM) and complex **1** or complex **3** (160 μM) in phosphate buffer (pH 7.2) at ambient temperature. The EPR spectra of the resulting solutions were recorded at various time intervals over 24 h.

^{1}H NMR of Reactions with Duroquinone. NADH (1.5 mol equiv) was added to an NMR tube containing a 2 mM solution of $[(\eta^5\text{-}C_5Me_5)Ir(phen)(H_2O)]^{2+}$ (**1**) in 10% MeOD-d_4/90% H_2O at ambient temperature. ^{1}H NMR spectra of the resulting solutions were recorded at 298 K. After 15 min of reaction, menadione (4 mM) was added to the above solution, and the ^{1}H NMR spectra of the resulting solutions were recorded at 298 K.

NADH (3 mol equiv) was added to an NMR tube containing a 1 mM solution of $[(\eta^5\text{-}C_5Me_4C_6H_5)Ir(phen)(H_2O)]^{2+}$ (**3**) in 10% MeOD-d_4/90% H_2O at ambient temperature. ^{1}H NMR spectra of the resulting solutions were recorded at 298 K. After 15 min of reaction, duroquinone (4 mM) was added to the above solution, and the ^{1}H NMR spectra of the resulting solutions were recorded at 298 K.

EPR of Reactions with Duroquinone. Duroquinone (2 mM) was added to a solution containing NADH (1 mM) and complex **1** or complex **3** (330 μM) in phosphate buffer (pH 7.2) at ambient temperature. The EPR spectra of the resulting solutions were recorded at various time intervals over a period of 24 h.

References

[1] T. L. Hwang, A. J. Shaka, *J. Magn. Reson.,Ser. A* **1995**, *112*, 275-279.

[2] U. B. F. Neese, D. Ganiouchine, S. Koßmann, T. Petrenko, C. Riplinger, F. Wennmohs, ORCA 2.9.1 ed.; University of Bonn: Bonn, 2012.

[3] a) J. P. Perdew, W. Yue, *Phys. Rev. B* **1986**, *33*, 8800-8802; b) A. D. Becke, *Phys. Rev. A* **1988**, *38*, 3098-3100.

[4] D. A. Pantazis, X.-Y. Chen, C. R. Landis, F. Neese, *J. Chem. Theory. Comput.* **2008**, *4*, 908-919.

[5] S. Grimme, *J. Comput. Chem.* **2006**, *27*, 1787-1799.

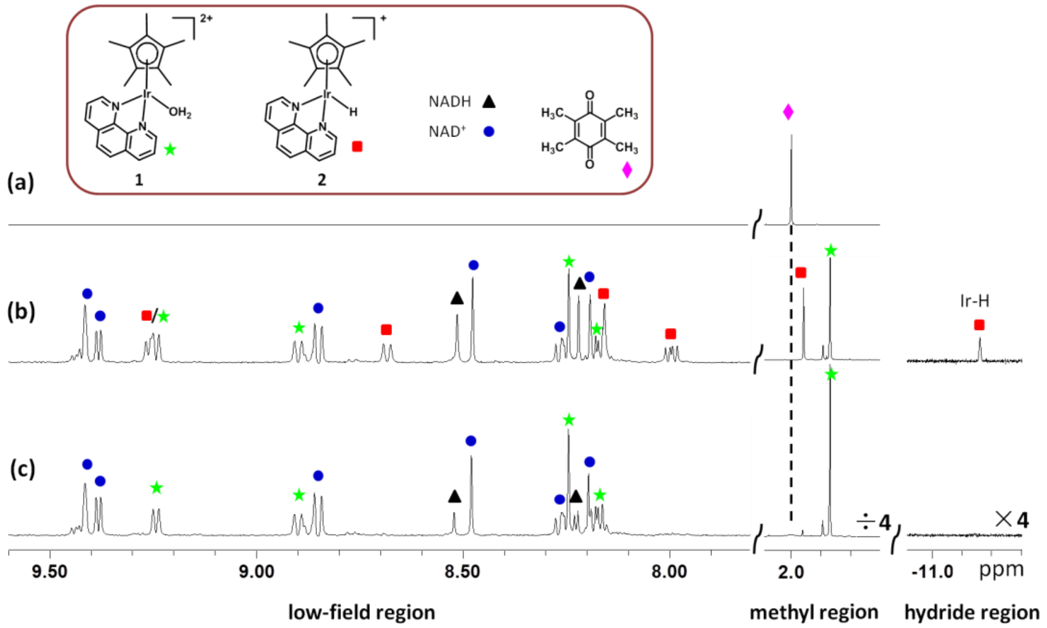

Figure S1. ^{1}H NMR spectra showing the formation of complex **2** $[(\eta^5\text{-}C_5Me_5)Ir(phen)(H)]^+$, followed by reaction with duroquinone in 10% MeOD-d_4/90% H_2O at 298 K. (a) Duroquinone as control; (b) generation of **2** produced by hydride transfer from NADH (3 mM) to complex **1** $[(\eta^5\text{-}C_5Me_5)Ir(phen)(H_2O)]^{2+}$ (2 mM); (c) addition of duroquinone (4 mM) results in the disappearance of the hydride peak after 10 min. The peak corresponding to duroquinone became broad after the reaction (indicated by the vertical line).

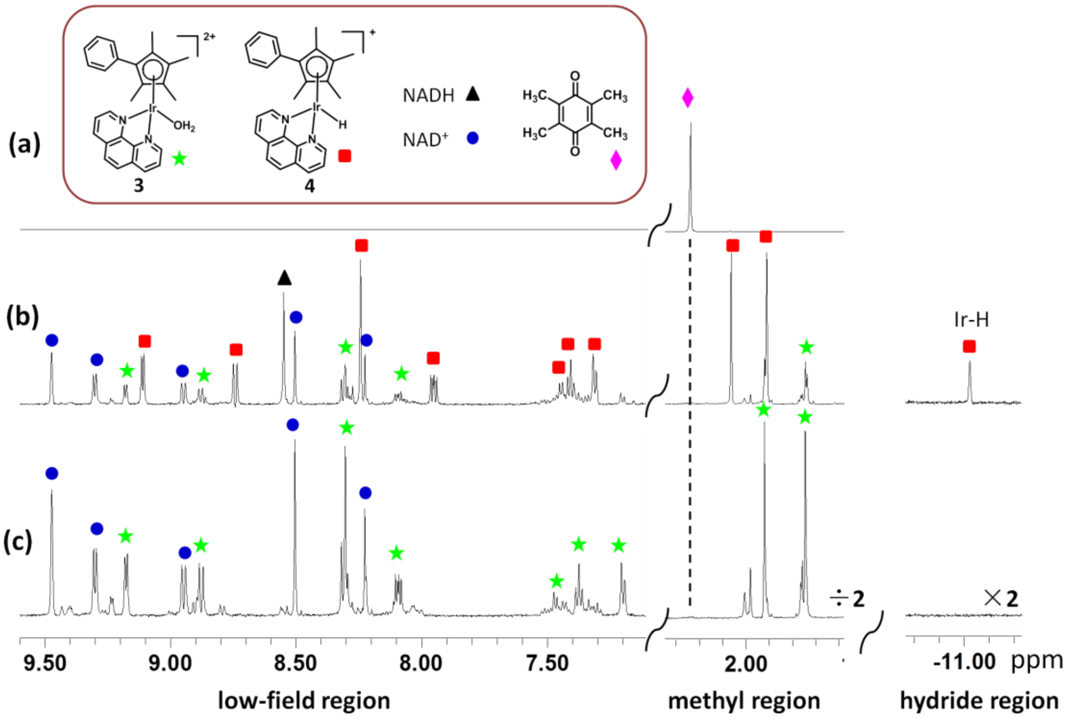

Figure S2. ^{1}H NMR spectra showing the formation of complex **4** $[(\eta^5\text{-}C_5Me_4C_6H_5)Ir(phen)(H)]^+$, followed by reaction with duroquinone in 10% MeOD-d_4/90% H_2O at 298 K. (a) Duroquinone as control; (b) generation of **4** produced by hydride transfer from NADH (3 mM) to complex **3** $[(\eta^5\text{-}C_5Me_4C_6H_5)Ir(phen)(H_2O)]^{2+}$ (1 mM); (c) addition of duroquinone (4 mM) results in the disappearance of the hydride peak after 10 min. Peaks corresponding to duroquinone became broad after the reaction, indicated by the black lines.

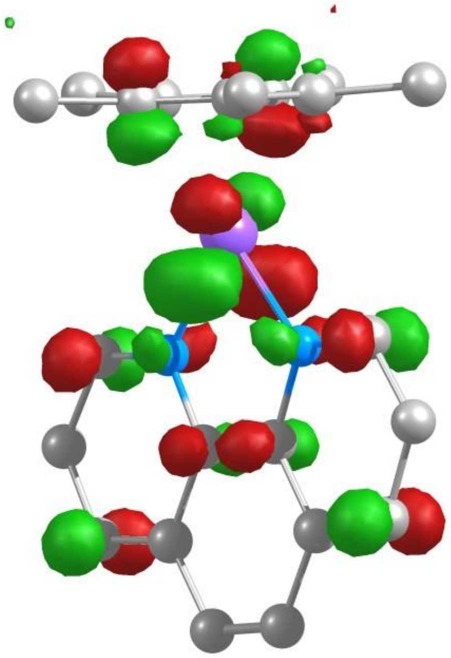

Figure S3. Plot of singly occupied MO for the Ir(II) complex in Scheme 2a.
